# Predicting outcome from subacute unresponsive wakefulness syndrome or vegetative state

**DOI:** 10.1186/cc13831

**Published:** 2014-04-15

**Authors:** Olivier Bodart, Steven Laureys

**Affiliations:** 1 Department of Neurology, University Hospital of Liege, Sart Tilman B35, avenue de l’hopital n°1, 4000 Liege, Belgium; 2 Coma Science Group, Cyclotron Research Centre, University of Liege, Sart Tilman B30, allee du 6 aout n°8, 4031 Liege, Belgium

## Abstract

Predicting recovery of consciousness in patients who survive their coma but evolve to a vegetative state (recently coined unresponsive wakefulness syndrome) remains a challenge. Most previous prognostic studies have focused on the acute coma phase. A novel outcome scale (combining behavioural, aetiology, electroencephalographic, sleep electroencephalographic and somatosensory evoked potential data) has been proposed for patients in subacute unresponsive wakefulness syndrome. The scale’s clinical application awaits validation in a larger population.

## 

The diagnosis, prognosis and therapeutic management of patients with disorders of consciousness remain a challenge. In a recent issue of *Critical Care*, Kang and colleagues proposed an innovative tool [1] to better assess the prognosis of patients with unresponsive wakefulness syndrome (UWS; previously vegetative state (VS) [2]). Studies on prognostication of patients with severe brain injuries, from anoxic-ischaemic, traumatic, or other aetiologies, have so far focused on the acute phase upon admission or during the patients stay in the ICU. Many of these studies have used mortality rate as an outcome (for example [3]) and their findings cannot be easily transposed to the population of subacute patients with VS/UWS.

Accurate outcome prediction is important for medical caregivers, families and healthcare administrators aiming to optimise the attribution of limited resources. Previous studies on subacute or chronic disorders of consciousness have shown that increased age at time of injury and decreased levels of consciousness are predictors of bad recovery in ischaemic-anoxic [4,5], subarachnoid haemorrhage [6] or traumatic [7] aetiologies. A limited number of studies also used electrophysiological measurements in the subacute or chronic phase with the aim of providing a more accurate prognosis, assessing electroencephalographic reactivity [8], the presence of N400 evoked potentials to speech [9] or the absence of the middle component of auditory evoked potentials [10]. Finally, functional magnetic resonance imaging studies in UWS have shown that high-level cortical activation heralds better outcome [11].

The recently proposed TMSEN outcome score, developed by Kang and colleagues [1], would allow clinicians to better estimate the probability of consciousness recovery in patients with post-coma VS/UWS. To the best of our knowledge, this is the first time a multi-item scale, combining information from clinical and complimentary testings, has been specifically designed to assess the prognosis in this challenging condition. The combination of five clinical and electrophysiological items - type of brain injury (traumatic vs. nontraumatic), flexion motor response to noxious stimuli, electroencephalographic reactivity, sleep spindles and N20 component of somatosensory evoked potentials - provided high positive and negative predictive values in the 56 enrolled patients [1], which now awaits validation in larger multicentric cohorts.

How easily this scale can be translated to real-life hospital settings, where long-duration overnight electroencephalographic recordings and even somatosensory evoked potentials are not always easily available, remains to be shown. The fact that no effect of age was found in the current study, as noted by the authors, might be related to the same small and heterogeneous sample size. Previous studies have found that older patients with severe brain injuries have worse outcome [4-7,10]. Information coming from structural (for example, diffusion tensor imaging) and functional magnetic resonance imaging, also assumed to have predictive power in coma [12,13], could be further assessed in the subacute and chronic setting and could possibly be added to future multimodal prognostic scales. Finally, it is important to stress that the use of these scales in terms of intensity of care, long-term management and end-of-life decisions needs a wider societal discussion involving not only physicians working in the ICU, neurology and rehabilitation, but also ethicists and legal scholars.

In conclusion, we very much welcome the authors’ effort aiming to reduce prognostic uncertainty in the challenging group of patients with VS/UWS [1] and emphasise that no good medical (and ethical) decisions can be made in the absence of good clinical and prognostic knowledge [14,15].

## Abbreviations

UWS: Unresponsive wakefulness syndrome; VS: Vegetative state.

## Competing interests

The authors declare that they have no competing interests.

## References

[B1] Kang X-G Li L Wei D Xu X-X Zhao R Jing Y-Y Su Y-Y Xiong L-Z Zhao G Jiang W Development of a simple score to predict outcome for unresponsive wakefulness syndrome Crit Care 2014 18 R37 10.1186/cc13745 24571596 PMC4056750

[B2] Laureys S Celesia GG Cohadon F Lavrijsen J Leon-Carrion J Sannita WG Sazbon L Schmutzhard E von Wild KR Zeman A Dolce G Unresponsive wakefulness syndrome: a new name for the vegetative state or apallic syndrome BMC Med 2010 8 68 10.1186/1741-7015-8-68 21040571 PMC2987895

[B3] Grmec S Gasparovic V Comparison of APACHE II, MEES and Glasgow Coma Scale in patients with nontraumatic coma for prediction of mortality Crit Care 2001 5 19 23 10.1186/cc973 11178221 PMC29052

[B4] Howell K Grill E Klein AM Straube A Bender A Rehabilitation outcome of anoxic-ischaemic encephalopathy survivors with prolonged disorders of consciousness Resuscitation 2013 84 1409 1415 10.1016/j.resuscitation.2013.05.015 23747956

[B5] Estraneo A Moretta P Loreto V Lanzillo B Cozzolino A Saltalamacchia A Lullo F Santoro L Trojano L Predictors of recovery of responsiveness in prolonged anoxic vegetative state Neurology 2013 80 464 470 10.1212/WNL.0b013e31827f0f31 23303855

[B6] Klein AM Howell K Straube A Pfefferkorn T Bender A Rehabilitation outcome of patients with severe and prolonged disorders of consciousness after aneurysmal subarachnoid hemorrhage (aSAH) Clin Neurol Neurosurg 2013 115 2136 2141 10.1016/j.clineuro.2013.08.004 23993657

[B7] Klein AM Howell K Vogler J Grill E Straube A Bender A Rehabilitation outcome of unconscious traumatic brain injury patients J Neurotrauma 2013 30 1476 1483 10.1089/neu.2012.2735 23477301 PMC3751265

[B8] Logi F Pasqualetti P Tomaiuolo F Predict recovery of consciousness in post-acute severe brain injury: the role of EEG reactivity Brain Inj 2011 25 972 979 10.3109/02699052.2011.589795 21745174

[B9] Steppacher I Eickhoff S Jordanov T Kaps M Witzke W Kissler J N400 predicts recovery from disorders of consciousness Ann Neurol 2013 73 594 602 10.1002/ana.23835 23443907

[B10] Luaute J Maucort-Boulch D Tell L Quelard F Sarraf T Iwaz J Boisson D Fischer C Long-term outcomes of chronic minimally conscious and vegetative states Neurology 2010 75 246 252 10.1212/WNL.0b013e3181e8e8df 20554940

[B11] Di H Boly M Weng X Ledoux D Laureys S Neuroimaging activation studies in the vegetative state: predictors of recovery? Clin Med (Northfield Il) 2008 8 502 507 10.7861/clinmedicine.8-5-502 PMC4953932 18975482

[B12] Galanaud D Perlbarg V Gupta R Stevens RD Sanchez P Tollard E de Champfleur NM Dinkel J Faivre S Soto-Ares G Veber B Cottenceau V Masson F Tourdias T Andre E Audibert G Schmitt E Ibarrola D Dailler F Vanhaudenhuyse A Tshibanda L Payen JF Le Bas JF Krainik A Bruder N Girard N Laureys S Benali H Puybasset L Assessment of white matter injury and outcome in severe brain trauma: a prospective multicenter cohort Anesthesiology 2012 117 1300 1310 10.1097/ALN.0b013e3182755558 23135261

[B13] Luyt CE Galanaud D Perlbarg V Vanhaudenhuyse A Stevens RD Gupta R Besancenot H Krainik A Audibert G Combes A Chastre J Benali H Laureys S Puybasset L Diffusion tensor imaging to predict long-term outcome after cardiac arrest Anesthesiology 2012 117 1311 1321 10.1097/ALN.0b013e318275148c 23135257

[B14] Giacino JT Fins JJ Laureys S Schiff ND Disorders of consciousness after acquired brain injury: the state of the science Nat Rev Neurol 2014 10 99 114 10.1038/nrneurol.2013.279 24468878

[B15] Jox RJ Bernat JL Laureys S Racine E Disorders of consciousness: responding to requests for novel diagnostic and therapeutic interventions Lancet Neurol 2012 11 732 738 10.1016/S1474-4422(12)70154-0 22814543

